# Evaluation of Gemini 2.0 AI in Classifying Breast Lesion Status From Dynamic Contrast-Enhanced MRI: A Preliminary Study

**DOI:** 10.7759/cureus.94144

**Published:** 2025-10-08

**Authors:** Nitin Chetla, Trisha Naidu, Shivam Patel, Andrew Bouras, Harshita Kacham, Vinisha Bonagiri, Nasif Zaman

**Affiliations:** 1 Medicine, University of Virginia School of Medicine, Charlottesville, USA; 2 Computational and Systems Neuroscience, Virginia Polytechnic Institute and State University, Blacksburg, USA; 3 Data Science, University of Virginia, Charlottesville, USA; 4 Osteopathic Medicine, Nova Southeastern University Dr. Kiran C. Patel College of Osteopathic Medicine, Clearwater, USA; 5 Medicine, Osmania University, Hyderabad, IND; 6 Medicine, Dr. NTR University of Health Sciences, Vijayawada, IND; 7 Computer Science and Engineering, University of Nevada, Reno, Reno, USA

**Keywords:** ai diagnostics, breast cancer, breast mri, contrast-enhanced mri, deep learning, gemini 2, lesion classification, medical imaging, prompt engineering, radiology ai

## Abstract

Introduction: Breast MRI, particularly dynamic contrast-enhanced (DCE) MRI, offers high sensitivity in detecting breast lesions but suffers from variability in interpretation. Artificial intelligence (AI) tools like Gemini 2.0 (Google AI, Mountain View, CA) may help streamline and improve diagnostic accuracy. This study evaluates Gemini 2.0’s performance in classifying breast lesion status using application programming interface (API)-based image analysis.

Methods: MRI images were sourced from the publicly available fastMRI Breast dataset, which includes axial DCE-MRI sequences acquired using a 3D Golden-angle Radial Sparse Parallel (GRASP) protocol. Images were converted from DICOM to PNG for compatibility with Gemini 2.0’s API. Two binary classification prompts were tested. Prompt 1 distinguished between (A) benign or malignant lesion and (B) negative lesion status using 100 patient scans. Prompt 2 classified (A) benign vs. (B) malignant lesions in a separate set of 180 patient scans. Responses from the Gemini 2.0 API were recorded, and performance was assessed using accuracy, precision, recall, and F1-score.

Results: For prompt 1, Gemini 2.0 achieved 50% accuracy. It identified all benign or malignant lesions (100% recall) but misclassified all negative cases, yielding 0% recall for negative lesion status. The F1-score was 0.67 for benign/malignant lesions and 0 for negative lesions. For prompt 2, the model achieved 52% accuracy. It exhibited 97% recall for malignant lesions but only 7% for benign lesions, reflecting a strong bias toward malignancy. The weighted average F1-score was 0.39.

Discussion: Gemini 2.0 shows promise in flagging lesion presence but lacks the discriminatory power needed to distinguish benign from malignant or negative cases reliably. The high false-positive rate and class imbalance indicate a need for algorithmic refinement and further validation. Larger and more diverse training datasets are necessary to improve performance and reduce bias. Future research should compare AI classifications to radiologist interpretations in a prospective setting to assess clinical utility.

Conclusion: Gemini 2.0 offers preliminary utility in breast lesion detection on DCE-MRI, particularly in identifying malignancy. However, its limited specificity and poor differentiation between lesion types underscore the need for continued development before clinical deployment.

## Introduction

Breast cancer represents one of the most prevalent cancers affecting women worldwide [1], with early detection significantly improving prognosis and survival outcomes. Breast magnetic resonance imaging (MRI), particularly dynamic contrast-enhanced (DCE) MRI, is increasingly employed for its superior sensitivity in identifying breast lesions compared to conventional imaging modalities [2]. However, accurately distinguishing between benign and malignant lesions remains clinically challenging [2] and heavily reliant on radiologist expertise. Artificial intelligence (AI)-driven tools offer the potential to augment diagnostic accuracy, reduce variability, and expedite clinical workflow. Unlike task-specific breast MRI models, Gemini 2.0 (Google AI, Mountain View, CA) is a general-purpose multimodal model not trained for radiology, and here it was used in a zero-shot setting. This study conducts a preliminary, proof-of-concept evaluation of Gemini 2.0’s capability in classifying breast lesion status from MRI images using prompts directed through its application programming interface (API).

## Materials and methods

The study utilized breast MRI images sourced from the publicly available fastMRI Breast dataset developed by NYU Langone Health [3], part of the broader fastMRI initiative [4,5]. Images were originally acquired as axial DCE MRI sequences using a 3D Golden-angle Radial Sparse Parallel (GRASP) acquisition protocol on 3T scanners. Lesion annotations (benign, malignant, negative) were taken directly from the dataset metadata provided by the fastMRI Breast release. No independent radiologist re-annotation was performed.

Inclusion criteria were (1) availability of a full DCE-MRI series in DICOM format, (2) clear lesion annotations indicating benign, malignant, or negative findings, and (3) adequate image quality without motion or reconstruction artifacts. Exclusion criteria included incomplete imaging series, missing diagnostic labels, or poor image quality (e.g., signal dropout or aliasing artifacts).

Each patient’s DICOM image series was deidentified and converted into PNG (Portable Network Graphics) format. During preprocessing, images were visually and programmatically checked for consistency in orientation and slice order. Series were then grouped into labeled cohorts based on diagnostic metadata, and two balanced datasets were constructed through stratified sampling by diagnostic label. This balancing was performed deliberately to ensure that class-wise performance could be interpreted, though it does not reflect real-world prevalence. Prompt 1 distinguished between “A) Benign or Malignant Lesion Status” and “B) Negative Lesion Status” using 100 patient scans (50 with lesions, 50 negative). Prompt 2 differentiated “A) Benign Lesion Status” from “B) Malignant Lesion Status” across 180 cases (90 benign, 90 malignant).

Prompt 1: “This is a series of Breast MRI images from a patient. Based on the images, what would the lesion status be? A) Benign or Malignant Lesion Status B) Negative Lesion Status. Answer with a single letter ONLY (‘A’ or ‘B’).”

Prompt 2: “This is a series of Breast MRI images from a patient. Based on the images, what would the lesion status be? A) Benign Lesion Status B) Malignant Lesion Status. Answer with a single letter ONLY (‘A’ or ‘B’).”

Each patient-level MRI series was submitted in a single API call, containing the full set of images for that case, and Gemini 2.0 returned one categorical label per case. We did not request or obtain per-slice scores or lesion-localization outputs. Responses were stored and mapped to ground-truth labels. No fine-tuning or task-specific retraining of Gemini 2.0 was performed; all inferences were conducted in zero-shot mode using the pre-deployed Gemini 2.0 Vision-Language model.

Classification performance was evaluated using standard metrics: accuracy, precision, recall, F1-score, and support. Confidence intervals for proportion-based metrics (e.g., accuracy, recall) were calculated using the Wilson score method, and confidence intervals for F1-scores were estimated via nonparametric bootstrapping at the case level.

## Results

**Figure 1:**
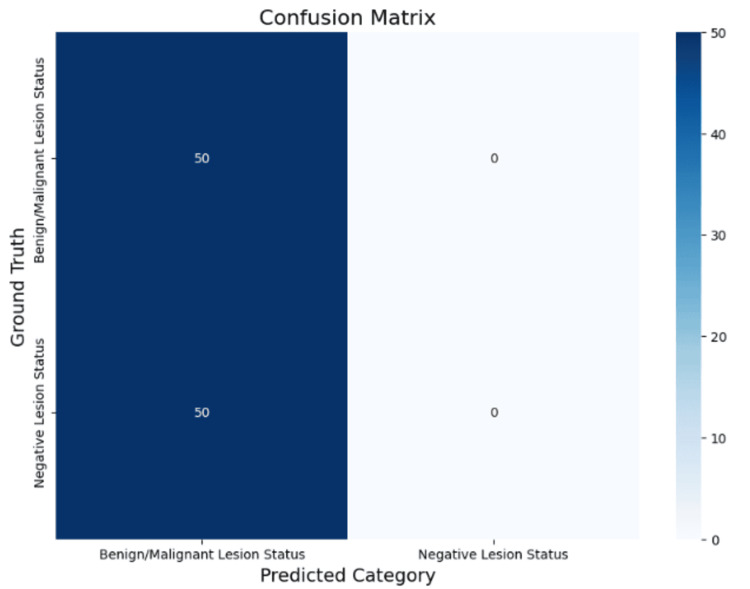
Confusion matrix for prompt 1 (benign/malignant lesion status vs. negative lesion status). Matrix visualization of Gemini 2.0 predictions under prompt 1. All 50 lesion-positive cases (benign or malignant) were correctly identified, while all 50 negative cases were misclassified as lesion-positive. This reflects perfect sensitivity (recall) for lesion-positive status but zero specificity for negative status.

For prompt 1 (Table 1 and Figure 1), Gemini 2.0 achieved an overall accuracy of 50%. Macro average precision, recall, and F1-score were 0.25, 0.50, and 0.33, respectively, while weighted averages were identical at 0.25, 0.50, and 0.33. Recall for benign or malignant lesions was perfect (100%), but all negative cases were misclassified as positive, resulting in zero recall for negative lesion status.

**Table 1:** Class-wise performance metrics for Gemini 2.0 under prompt 1 (benign/malignant vs. negative lesion status). Values represent precision, recall, F1-score, and support for each class. Accuracy is reported separately. Macro and weighted averages summarize overall model performance across classes.

Class	Precision	Recall	F1-score	Support
Benign/malignant lesion status	0.50	1.00	0.67	50
Negative lesion status	0.00	0.00	0.00	50

**Figure 2:**
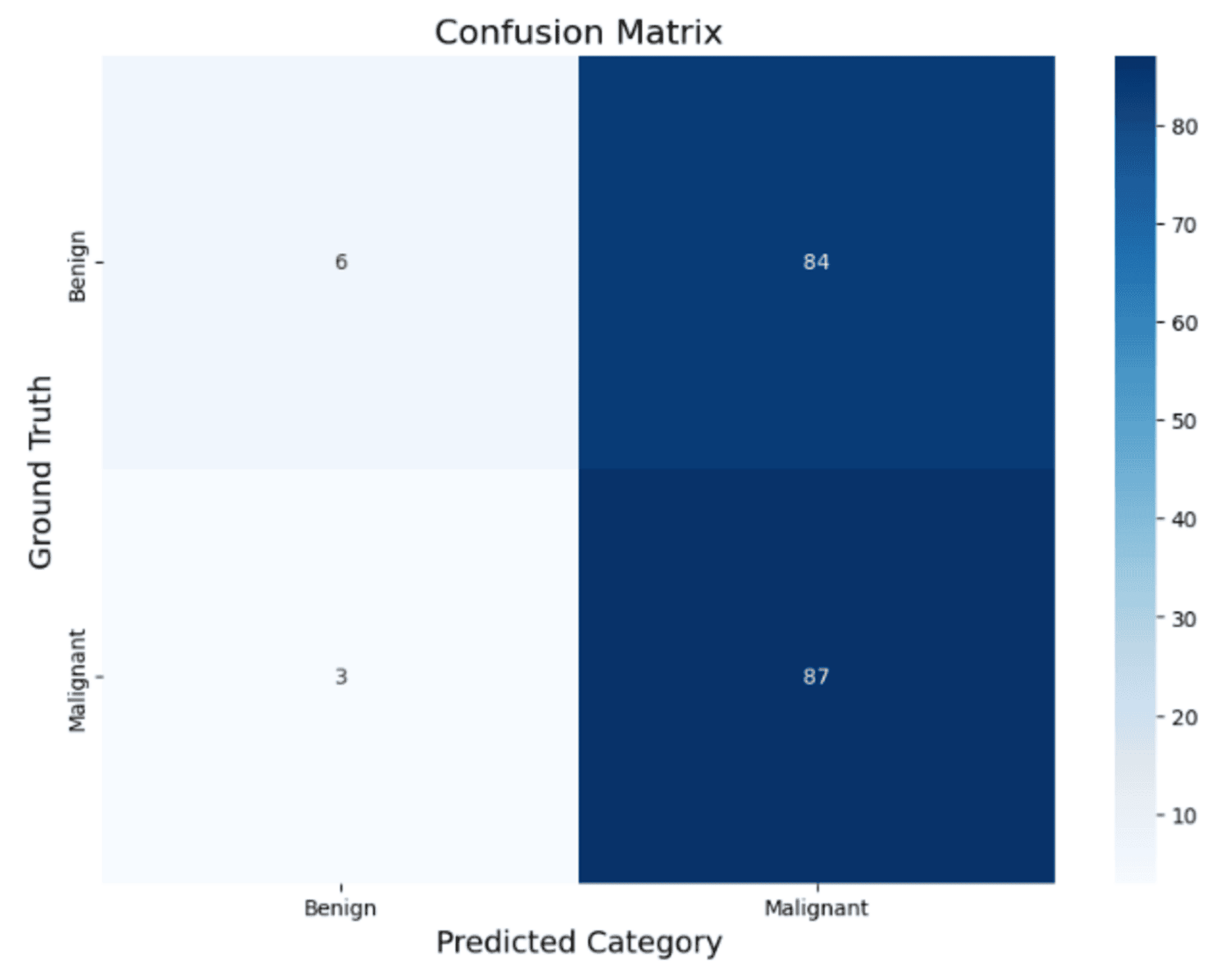
Confusion matrix for prompt 2 (benign lesion status vs. malignant lesion status). Matrix visualization of Gemini 2.0 predictions under prompt 2. The model correctly identified 87 malignant cases but misclassified 84 benign cases as malignant. This illustrates high sensitivity for malignant lesions but very low sensitivity for benign lesions, indicating a strong bias toward malignancy.

For prompt 2 (Table 2 and Figure 2), Gemini 2.0 achieved an overall accuracy of 52%. Macro averages for precision, recall, and F1-score were 0.59, 0.52, and 0.39, respectively, with identical weighted averages. The model showed high recall for malignant lesions (97%) but very low recall for benign lesions (7%), indicating a bias toward malignant classification.

**Table 2:** Class-wise performance metrics for Gemini 2.0 under prompt 2 (benign vs. malignant lesion status). Values represent precision, recall, F1-score, and support for each class. Accuracy is reported separately. Macro and weighted averages are provided to summarize model performance across both classes.

Class	Precision	Recall	F1-score	Support
Benign lesion status	0.67	0.07	0.12	90
Malignant lesion status	0.51	0.97	0.67	90

**Figure 3:**
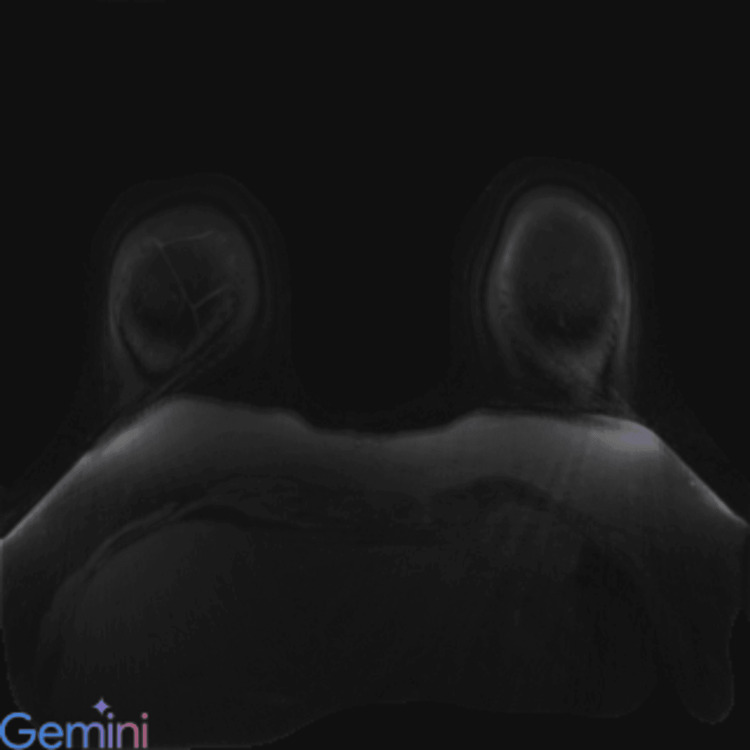
Gemini 2.0 application programming interface (API) output for prompt 1 – Example case showing classification as benign or malignant lesion status. Representative slice from a lesion-positive patient series, though the lesion may not be visible on the displayed slice. Ground-truth labels were derived from the fastMRI Breast dataset metadata. Gemini 2.0 provided only case-level outputs without lesion localization or bounding box overlays. The model’s response to prompt 1 returned classification “A.”

**Figure 4:**
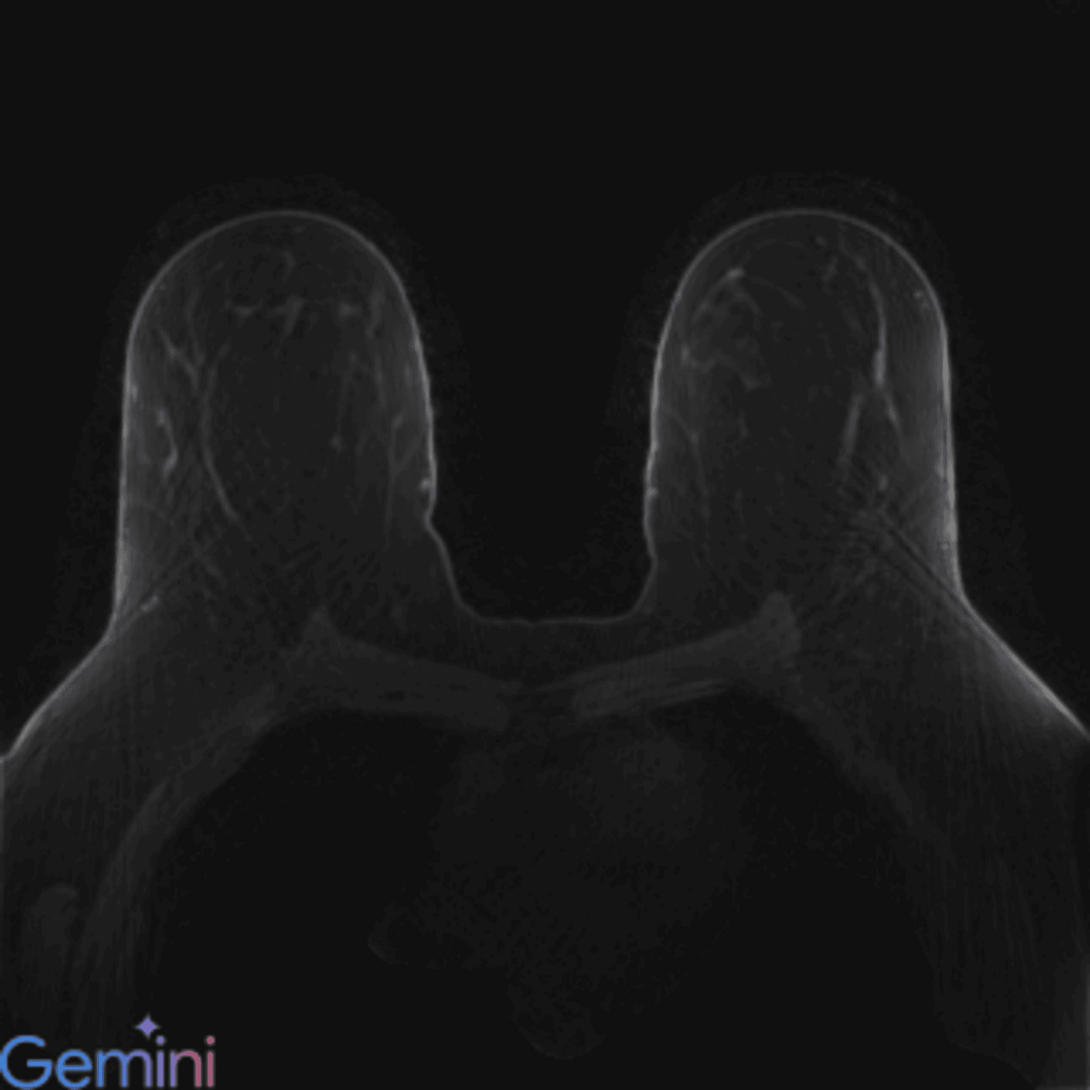
Gemini 2.0 application programming interface (API) output for prompt 2 – Example case showing classification as benign lesion status. Representative slice from a benign case, though the lesion may not be visible on the displayed slice. Ground-truth labels were taken from the fastMRI Breast dataset metadata. Gemini 2.0 returned only case-level categorical outputs without lesion localization. The model’s response to prompt 2 returned classification “A.”

**Figure 5:**
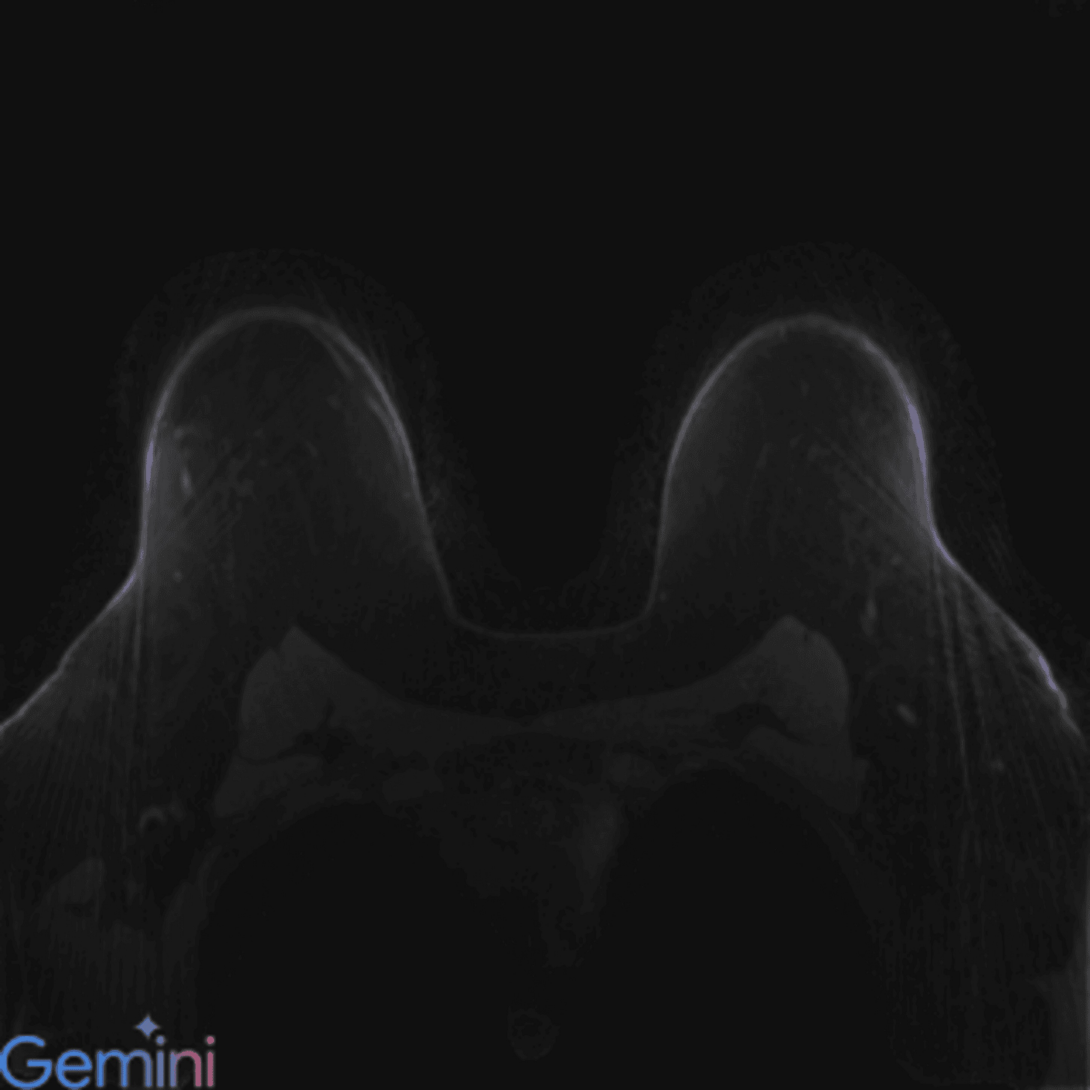
Gemini 2.0 application programming interface (API) output for prompt 2 – Example case showing classification as malignant lesion status. Representative slice from a malignant case, though the lesion may not be visible in the displayed slice. Ground-truth labels were obtained from the fastMRI Breast dataset metadata. Gemini 2.0 returned only case-level outputs without lesion localization or overlays. The model’s response to prompt 2 returned classification “B.”

Table 3 provides representative Gemini 2.0 outputs consistent with the confusion matrices, illustrating correct and incorrect classifications across lesion-positive cases in prompt 1 and benign/malignant cases in prompt 2. No correctly classified negatives were observed in prompt 1 (Figures 3-5).

**Table 3:** Example of Gemini 2.0 responses for selected cases. Examples shown are consistent with the confusion matrices. No pixel-level annotations or overlays were available from the application programming interface (API). Gemini returned only case-level categorical outputs.

Figure	Image file	Prompt used	Classification example
Figure 3	slice_013_frame_003.png	Prompt 1 – "A) Benign or Malignant Lesion Status" vs. "B) Negative Lesion Status"	Classified as A: Benign or Malignant Lesion Status
Figure 4	slice_083_frame_003.png	Prompt 2 – "A) Benign Lesion Status" vs. "B) Malignant Lesion Status"	Classified as A: Benign Lesion Status
Figure 5	slice_132_frame_001.png	Prompt 2 – "A) Benign Lesion Status" vs. "B) Malignant Lesion Status"	Classified as B: Malignant Lesion Status

## Discussion

Model performance overview

Gemini 2.0, a general-purpose multimodal model not trained on breast imaging, shows potential as an adjunct tool in breast MRI lesion classification. This proof-of-concept study demonstrated perfect recall (1.00) for lesion-positive cases in prompt 1, indicating high sensitivity, but failed to identify any true negatives, resulting in an overall accuracy of 0.50. In prompt 2, designed to distinguish benign from malignant lesions, Gemini achieved very high recall for malignant cases (0.97) but extremely poor recall for benign lesions (0.07). This imbalance suggests a systematic bias toward malignancy, reflecting the limitations of applying a general-purpose model in a zero-shot setting without domain-specific adaptation.

Background on Gemini 2.0

Gemini 2.0 is a general-purpose vision-language model that accepts multiple images and a text prompt and returns a case-level categorical response. In the API configuration used here, Gemini provided only a single label per patient series; it did not output pixel-level heatmaps, bounding boxes, or lesion localization. This contrasts with task-specific radiology AI systems, which typically operate at the voxel or slice level and provide interpretable overlays.

Comparison with primary research studies

These findings align with prior primary studies that used deep learning on breast MRI. Wu et al. developed a dual-branch deep learning fusion convolutional neural network (CNN) trained end-to-end on DCE-MRI and achieved high diagnostic accuracy with improved specificity [6]. Chen et al. implemented a Faster R‑CNN-based model on DCE-MRI data with external validation and reported a sensitivity of 96.2% and an area under the curve (AUC) of 0.955 for detecting lesions, outperforming many traditional CNN-based classifiers [7]. Li et al. combined kinetic curve features with radiomic features from DCE-MRI in a fully automated pipeline and achieved an AUC of 0.94 on a dataset of 200 DCE-MRI cases (172 benign, 126 malignant) [8]. These task-specific, pixel-level models outperform Gemini’s zero-shot performance, particularly in benign vs. malignant differentiation.

Study limitations

This study has several limitations. First, the datasets were modest in size (100 and 180 cases) and deliberately balanced by class, which does not reflect real-world prevalence and may bias operating points. Second, only axial GRASP-based DCE-MRI sequences were used; other sequences, such as diffusion-weighted imaging (DWI) and T2-weighted imaging, which improve specificity, were omitted. Third, no clinical metadata (e.g., Breast Imaging-Reporting and Data System (BI-RADS) category, patient age, or history) was included, unlike clinical practice, where such context is crucial. Fourth, labels were taken from dataset metadata without independent radiologist re-annotation, and no radiologist benchmark was included, limiting the interpretability of comparative performance. Fifth, the evaluation relied solely on forced-choice prompts with case-level outputs; Gemini 2.0 did not provide lesion localization or pixel-level explanations, reducing clinical trust. Finally, this was a single-split, proof-of-concept analysis without cross-validation or external validation, limiting reproducibility and generalizability.

Clinical implications

The observed false-positive bias has potential clinical consequences. A tendency to over-call malignancy could increase call-backs, additional imaging, and biopsies, contributing to patient anxiety and increased workload for radiologists. In current workflows, radiologists integrate multiparametric MRI and clinical metadata to assign BI-RADS categories that balance sensitivity and specificity. Any AI tool with a high false-positive rate would therefore need careful operating-point calibration and direct benchmarking against radiologist performance before consideration for clinical deployment.

Implications and future directions

Future studies should fine-tune or retrain Gemini-like models on labeled breast MRI datasets that include multiparametric sequences (DWI, T2-weighted) and relevant clinical metadata. Incorporating kinetic curve information, as Li et al. demonstrated, may improve benign versus malignant discrimination. External validation across scanner types and acquisition protocols is needed to support generalizability. Most importantly, prospective studies comparing AI outputs with radiologist BI-RADS assessments are required to determine whether such systems can add value in real-world workflows.

## Conclusions

Gemini 2.0 demonstrates preliminary utility as a general-purpose AI model applied in a zero-shot setting for breast lesion evaluation on DCE MRI. Its high recall for malignant lesions suggests potential value in flagging suspicious cases, but its inability to reliably identify negative findings or distinguish benign lesions underscores its current limitations. The observed false-positive bias and class imbalance highlight the need for domain-specific training, incorporation of multiparametric MRI and clinical metadata, and improved interpretability. Rigorous validation in real-world clinical settings, including direct comparison with radiologist BI-RADS performance, is essential before such models can be considered for clinical integration.
